# Diligent Medical Activities of a Publicly Designated Medical Institution for Infectious Diseases Pave the Way for Overcoming COVID-19: A Positive Message to People Working at the Cutting Edge

**DOI:** 10.1093/cid/ciaa694

**Published:** 2020-05-31

**Authors:** Tatsuya Nagano, Jun Arii, Mitsuhiro Nishimura, Naofumi Yoshida, Keiji Iida, Yoshihiro Nishimura, Yasuko Mori

**Affiliations:** 1 Division of Respiratory Medicine, Department of Internal Medicine, Kobe University Graduate School of Medicine, Kobe, Hyogo, Japan; 2 Division of Clinical Virology, Center for Infectious Diseases, Kobe University Graduate School of Medicine, Kobe, Hyogo, Japan; 3 Division of Cardiovascular Medicine, Department of Internal Medicine, Kobe University Graduate School of Medicine, Kobe, Hyogo, Japan; 4 Division of Diabetes and Endocrinology, Hyogo Prefectural Kakogawa Medical Center, Kakogawa, Hyogo, Japan

To the Editor—Severe acute respiratory syndrome coronavirus 2 (SARS-CoV-2), which causes coronavirus disease 2019 (COVID-19), has spread worldwide [1]. The possibility of virus transmission from patients with COVID-19 to medical staff is of primary concern. Recently, Htun et al [2] performed hospital-wide fever and sickness surveillance for 1524 medical staff working in COVID-19 areas and showed that all medical staff who suffered from fever were negative for SARS-CoV-2 by polymerase chain reaction (PCR) test. However, screening for COVID-19 in medical staff without fever is also important, since the asymptomatic proportion of COVID-19 cases was estimated to be 17.9% [3]. Therefore, on 1, 7, and 8 May 2020 we collected sera from 509 healthy medical staff members working to treat patients with COVID-19 at the Hyogo Prefectural Kakogawa Medical Center, which has 353 beds and is 1 of 55 publicly designated medical institutions for infectious diseases—including Ebola, smallpox, plague, tuberculosis, severe acute respiratory syndrome (SARS), and Middle East respiratory syndrome (MERS)—in Japan. Immunoglobulin G (IgG) antibodies for SARS-CoV-2 in each serum sample were analyzed by immunochromatographic test (2019-nCoV Ab Test; INNOVITA, Hebei, China), which includes colloidal gold coated with spike and nucleocapsid protein of SARS-CoV-2 as a tracer. The mean number of hospitalized patients with COVID-19 was 20 (95% confidence interval, 18–22). The 509 medical staff members consisted of 88 men and 421 women with a median age of 39 (range, 18–66) years. They were 77 doctors, 310 nurses, 1 pharmacist, 20 radiology technicians, 19 laboratory medical technologists, and 82 medical assistants. A total of 115, 18, and 72 worked in the intensive care unit, the ambulatory unit for patients with fever, and the ward for patients with COVID-19, respectively. The mean time from contact with patients with COVID-19 to sera collection was 24 days (95% confidence interval, 23–25 days). None of the medical staff in the hospital had IgG antibodies for SARS-CoV-2, whereas sera from patients with COVID-19 in the hospital, which were used as a positive control, showed a 100% (10/10) positive rate for IgG, suggesting the high specificity of the immunochromatographic test used here. In addition, the time-series behavior of IgG in sera from these patients was examined and is shown in Figure 1. Taken together, these results indicate that transmission from patients to medical staff did not occur in these medical staff members, and the standard preventive measures against infectious diseases can prevent SARS-CoV-2 exposure in medical staff.

**Figure 1.**
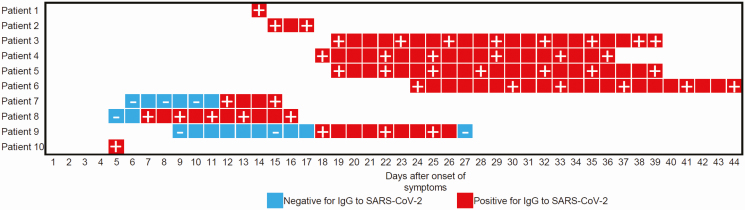
The time-series behavior of IgG in sera from patients with COVID-19 by immunochromatographic test (INNOVITA, Hebei, China). “+” or “−“ indicates positive or negative IgG for SARS-CoV-2 at each time point. The symptom onset date was reported from each patient. Abbreviations: COVID-19, coronavirus disease 2019; IgG, immunoglobulin G; SARS-CoV-2, severe acute respiratory syndrome coronavirus 2.

Despite the hard work of the brave medical workers around the world, many patients continue to die of COVID-19 [4]. Unfortunately, not just a few medical staff members have also died from nosocomial infections of SARS-CoV-2. The medical staff at the Kakogawa Medical Center have been in contact with patients with COVID-19 for up to 53 days, but so far there have been no incidents suspicious for nosocomial infection. Our surprising results suggest that standard preventive measures, if strictly followed, can prevent SARS-CoV-2 exposure in medical practitioners. Although all medical workers naturally fear SARS-CoV-2 exposure, we believe the current results could help alleviate their anxiety, and could provide courage and inspiration for their fight against COVID-19.

## Notes

***Author contributions***. T. N. and Y. M. had full access to all of the data in the study and take responsibility for the integrity of the data and the accuracy of the data analysis. Concept and design: T. N., Y. N., and Y. M. Acquisition, analysis, or interpretation of data: T. N., J. A., M. N., N. Y., K. I., Y. N., and Y. M. Drafting of the manuscript: T. N., J. A., M. N., and Y. M. Critical revision of the manuscript for important intellectual content: T. N., J. A., M. N., N. Y., K. I., Y. N., and Y. M. Statistical analysis: T. N., J. A., and M. N. Administrative, technical, or material support: J. A. and M. N. Supervision: K. I., Y. N., and Y. M.

***Acknowledgments.*** The authors thank Kazuro Sugimura, MD, PhD (Executive Vice President, Kobe University), for his full support in promoting this study. Yukiya Kurahashi, Lidya Handayani Tjan, Zhenxiao Ren, Anna Lystia Poetranto, Salma Aktar, Jing Rin Huang, and Silvia Sutandhio (Division of Clinical Virology, Center for Infectious Diseases, Kobe University Graduate School of Medicine) supported the immunochromatographic test. We express our sincere gratitude for the cooperation and participation of staffs of Hyogo Prefectural Kakogawa Medical Center.

***Financial support.*** This study was partly supported by the Hyogo Prefectural Government.

***Potential conflicts of interest.*** The authors: No reported conflicts of interest. All authors have submitted the ICMJE Form for Disclosure of Potential Conflicts of Interest.
